# Elementary and Practical Treatise of Internal Pathology

**Published:** 1845-09-01

**Authors:** 


*Elementary and Practical Treatise of Internal Pathology. By Grisolle.*—This is the title of a recent French treatise by A. Grisolle, D. M. P., Paris; an analytical review of which is contained in the July 1845 No. of the American Journal of Med. Sciences. The work (as appears from the review) is, in fact, a work on Practical Medicine, embodying the pathological and therapeutical principles at the present time current with the most approved of the French authorities. Works of this kind (called, with us, works on Practice) have for a long time been common in England and America, and their convenience and utility have been abundantly exemplified. It appears, however, that they are novel in France, this being the second of the kind which has been published. Their medical literature consists of monographs, treatises confined to special departments; and their dictionaries and encyclopædias, which are too voluminous and elaborate for general use. A compendious digest of approved French pathology and practice, would (if we may judge from our own wishes,) prove a very acceptable volume to the profession in this country. It would supply to those of us who are obliged to stay at home, and confine ourselves to the routine duties of our profession, much of that information, which, of late years, a goodly portion of those who are more fortunate, have deemed of sufficient interest and importance to traverse the ocean and gather at its sources. The author of the new work referred to, is well known in this country from his investigations of Pneumonitis. He has the reputation of being a close and accurate observer, careful in his analyses and inductions, and a man of strict professional integrity—a quality which is not in all instances the companion of great abilities and distinguished official position. In addition to these recommendations, he enjoys every opportunity for personal investigations in the large hospitals of Paris, and of becoming conversant with the doctrines and methods of the most eminent in the profession. These circumstances afford a guaranty that the work which he has published, is, what it professes to be, a correct representation of modern French pathology and practice.

We agree with the Reviewer in wishing that it may be translated, but not with additions as he suggests. We protest against the present custom of loading editions of works by cotemporaneous writers with annexations, excepting in certain cases in which the propriety and usefulness of this course areobvious. Let us have the work, if we are to have it, (as we hope we may) precisely as its author intended us to have it, without any parasitical appendages.

